# Defining the Interactions and Role of DCAF1/VPRBP in the DDB1-Cullin4A E3 Ubiquitin Ligase Complex Engaged by HIV-1 Vpr to Induce a G_2_ Cell Cycle Arrest

**DOI:** 10.1371/journal.pone.0089195

**Published:** 2014-02-18

**Authors:** Francine C. A. Gérard, Ruifeng Yang, Bizhan Romani, Alexis Poisson, Jean-Philippe Belzile, Nicole Rougeau, Éric A. Cohen

**Affiliations:** 1 Institut de Recherches Cliniques de Montréal (IRCM), Montréal, Québec, Canada; 2 Department of Microbiology, Infectiology and Immunology, Université de Montréal, Montréal, Québec, Canada; Institut National de la Santé et de la Recherche Médicale, France

## Abstract

HIV viral protein R (Vpr) induces a cell cycle arrest at the G_2_/M phase by activating the ATR DNA damage/replication stress signalling pathway through engagement of the DDB1-CUL4A-DCAF1 E3 ubiquitin ligase via a direct binding to the substrate specificity receptor DCAF1. Since no high resolution structures of the DDB1-DCAF1-Vpr substrate recognition module currently exist, we used a mutagenesis approach to better define motifs in DCAF1 that are crucial for Vpr and DDB1 binding. Herein, we show that the minimal domain of DCAF1 that retained the ability to bind Vpr and DDB1 was mapped to residues 1041 to 1393 (DCAF1 WD). Mutagenic analyses identified an α-helical H-box motif and F/YxxF/Y motifs located in the N-terminal domain of DCAF1 WD that are involved in exclusive binding to DDB1. While we could not identify elements specifically involved in Vpr binding, overall, the mutagenesis data suggest that the predicted β-propeller conformation of DCAF1 is likely to be critical for Vpr association. Importantly, we provide evidence that binding of Vpr to DCAF1 appears to modulate the formation of a DDB1/DCAF1 complex. Lastly, we show that expression of DCAF1 WD in the absence of endogenous DCAF1 was not sufficient to enable Vpr-mediated G_2_ arrest activity. Overall, our results reveal that Vpr and DDB1 binding on DCAF1 can be genetically separated and further suggest that DCAF1 contains determinants in addition to the Vpr and DDB1 minimal binding domain, which are required for Vpr to enable the induction of a G_2_ arrest.

## Introduction

Human immunodeficiency virus type 1 (HIV-1) encodes several accessory proteins – Vif, Vpr, Vpu, and Nef – that have been implicated in the modulation of the host environment to promote viral replication and evasion of innate and acquired immunity. One strategy used by these viral proteins to fulfill their functions is to usurp the host-ubiquitin machinery to mark host factors or antiviral proteins for proteasomal or lysosomal degradation [1], [2]. Hence, viral protein R (Vpr) was shown to engage a cullin 4A-ring E3 ubiquitin ligase (CRL4A) composed of cullin4A (CUL4A), the E3 ligase Roc1, damaged DNA binding protein 1 (DDB1) and a member of the DCAF (DDB1–cullin4-associated-factor) family called DCAF1 or VprBP (viral protein R binding protein) [3], [4], [5], [6], [7], [8], [9]. One unique feature of this family of multi-subunit E3 ubiquitin ligase is the presence of a three-β-propeller DDB1 adaptor whose role is to bridge the catalytic core organized on the CUL4A scaffold to the WD40-containing DCAF subunit, which acts as a substrate specificity receptor [10]. While biochemical and genetic evidence suggests that Vpr recruits the CRL4A (DCAF1) complex by making contact with the substrate recognition receptor DCAF1 [3], [4], [6], [7], [8], the identity of the host protein(s) targeted by the Vpr/CRL4A (DCAF1) E3 ligase complex has remained elusive.

Among the many biological functions attributed to HIV-1 Vpr, promotion of a cell cycle arrest at the G_2_/M phase was found dependent on the recruitment of CRL4A (DCAF1) [11]. Indeed, the formation of a Vpr/CRL4A (DCAF1) complex is required to establish an intracellular environment that mimics a DNA damage/replication stress response initiated by the ATR (Ataxia Telangectasia and Rad3-related) kinase in dividing cells, a process that ultimately leads to a G_2_ cell cycle arrest [12], [13], [14], [15]. Hence, by analogy with how other lentiviral accessory proteins usurp host E3 ubiquitin ligases, it was proposed that Vpr would recruit the CRL4A (DCAF1) E3 ligase to trigger poly-ubiquitination and subsequent degradation of a putative cellular protein(s) resulting in the activation of a G_2_ checkpoint. Indeed, we previously documented that Vpr could induce the lysine 48-linked poly-ubiquitination and proteasomal degradation of associated cellular protein(s) and that these processes were necessary for the activation of the ATR pathway [15]. Furthermore, our immunolocalization and subcellular fractionation studies revealed that Vpr interacted with DCAF1 within mobile nuclear structures that were associated with chromatin, suggesting that the Vpr-CRL4A (DCAF1) E3 ligase complex might target a yet-unknown chromatin-associated substrate(s) [16]. However, whether Vpr acts as an adaptor to recruit a new substrate(s) to the CRL4A (DCAF1) E3 ligase for ubiquitination and proteasomal degradation or whether Vpr enhances the ubiquitination of a natural substrate(s) targeted by DCAF1 remains unresolved.

The CRL4A is a widely expressed family of E3 ligases that are involved in regulating a diverse set of cellular processes, including development, transcription, replication and DNA repair [17]. Interestingly, Vpr and its paralog, Vpx, which is exclusively encoded by HIV-2 and several SIV lineages, are not the only viral proteins to usurp CRL4A E3 ligases [11], [18], [19]. Indeed, structural studies have provided key insights into how other viral proteins interact with the DDB1-CRL4A E3 ligase despite their sequence divergence. For instance, proteins V and X encoded by paramyxovirus simian virus 5 (SV5) and hepatitis B virus (HBV), respectively, interact directly with DDB1 to redirect the DDB1-CRL4A E3 ligase towards a new substrate [20]. Binding of these viral hijackers to DDB1 was mediated through a common α-helical structural element, also called H-box motif, which interacted directly with the surface of DDB1 ß-propeller C. Importantly, this structural element was found to be shared by various cellular DCAFs, suggesting that it might also play a role in anchoring cellular substrate-recruiting adaptors to CRL4A E3 ligase complexes [21].

DCAF1 was identified more than a decade ago as a Vpr-binding protein [22]. This 1507-amino acid substrate specificity receptor of the DDB1-CUL4A E3 ligase harbours several discrete domains. A central LisH (LIS1 homology) motif was recently identified as an oligomerization motif of the protein and the CRL4A complex and further shown to enhance the functional activity of the CRL4A (DCAF1) E3 ligase *in vitro* [23]. Interestingly, DCAF1 also contains a C-terminal region harbouring two WD-40 motifs, which was reported to be involved in the binding of both DDB1 and Vpr [6], [22], [24].

Although engagement of CRL4A (DCAF1) by Vpr was shown to be dependent on a physical interaction with DCAF1, the details of the interactions with the E3 ligase complex are still not fully understood. In this study, we sought to delineate determinants of DCAF1 that are crucial for DDB1 and/or Vpr binding to better understand the architecture of the putative DDB1-DCAF1-Vpr substrate-recognition module. Herein, we identified the molecular and structural determinants of DCAF1 implicated in DDB1 binding and showed that these domains are distinct from those involved in Vpr binding. Furthermore, we provide evidence that the role of DCAF1 in the Vpr-CRL4A (DCAF1) complex is not limited to bridging Vpr onto the DDB1-CUL4A E3 ligase, suggesting that DCAF1 might supply additional functions that would be central to Vpr-mediated G_2_ arrest.

## Materials and Methods

### Cells, antibodies and other reagents

HEK293T and HeLa cells were cultured as previously described [25]. The anti-HA (clone 12CA5) and anti-Myc (clone 9E10) monoclonal antibodies were produced from hybridomas obtained from the American Type Culture Collection. The following commercially available antibodies were used: rabbit anti-actin (Sigma-Aldrich), rabbit anti-DCAF1 (Protein-Tech), and rabbit anti-DDB1 (Santa Cruz Biotechnology), rabbit anti-DCAF1 (Accurate Chemical and Scientific Corporation) for confocal microscopy detection, rabbit anti-GAPDH (Cell Signalling Technology), rabbit anti-histone H3 (Abcam). The fluorochrome-conjugated antibodies were obtained from Molecular Probes (Invitrogen). DAPI (4′,6-Diamidino-2-phenylindole) was purchased from Sigma-Aldrich.

### Plasmids

The SVCMV-HA-Vpr plasmid and the plasmid encoding full-length myc-tagged DCAF1 (pCMV-myc-VPRBP also designated pCMV-myc-DCAF1) were described previously [8], [25]. The plasmid encoding myc-tagged DCAF1 WD 1041-1393 (pCS2-myc_6_-DCAF1 WD WT) was kindly provided by Dr. Florence Margottin (Institut Cochin, Paris, France). All DCAF1 WD mutants were generated by site-directed mutagenesis (Quick Change II Site-Directed Mutagenesis Kit, Agilent Technology) with the primers and template described in Supplemental Table 1. To this end, we generated twelve DCAF1 WD mutants, namely: pCS2-myc_6_-DCAF1 1041-1377, pCS2-myc_6_-DCAF1 WD L1054P, pCS2-myc_6_-DCAF1 WD R1057E, pCS2-myc_6_-DCAF1 WD R1247A, pCS2-myc_6_-DCAF1 WD R1283A, pCS2-myc_6_-DCAF1 WD R1247A/R1283A, pSC2-myc_6_-DCAF1 F1060A/Y1063A, pCS2-myc_6_-DCAF1 WD F1077A/F1080A, pCS2-myc_6_-DCAF1 Y1120A/F1123A, pCS2-myc_6_-DCAF1 WD Y1181A/F1184A, pCS2-myc_6_-DCAF1 WD F1255A/F1258A and pCS2-myc_6_-DCAF1 WD F1334A/F1337A. GFP-expressing plasmid pQBI-25 was obtained from Qbiogene (Carlsbad). To construct the plasmid encoding untagged full-length DCAF1 resistant to siRNA bp3 (pDCAF1-bp3R), an *Eco*R1/*Not*1 fragment encoding untagged full-length DCAF1 with synonymous substitution mutations at positions 3021, 3024, 3027, 3030, 3033 and 3036 (+1 represents the ATG initiation codon) was inserted into the pECFP-Ni vector (Clontech) linearized with the same restriction enzymes. Mutations conferring resistance to bp3 siRNA were generated by site directed mutagenesis (Quick Change II XL Site-Directed Mutagenesis Kit, Agilent Technology) using the following primers 5′-CCTTCCCCACCTACGCTGGACAGTATAATAACGGAATACCTGAGGGAACAACATGCTCGCTGCAAGAATCCAGTTGCCACCTGCCCACC-3′ and 5′-GGTGGGCAGGTGGCAACTGGATTCTTGCAGCGAGCATGTTGTTCCCTCAGGTATTCCGTTATTATACTGTCCAGCGTAGGTGGGGAAGG-3 and WT DCAF1 as template.

### Transfection, immunoprecipitation and western blot analysis

For analysis of protein complexes, HEK293T cells were transfected using the calcium phosphate precipitation method. Forty-eight hours later, cells were harvested, washed and lysed in Triton lysis buffer (50 mM Tris-HCl pH 7.5, 150 mM NaCl, 0.5% Triton X-100, and EDTA-free complete protease inhibitors (Roche Diagnosis)). Anti-Myc immunoprecipitation (IP) was performed using anti-Myc antibody (clone 9E10) followed by a purification step on protein A-conjugated Sepharose beads (VWR), whereas anti-HA immunoprecipitation was performed using 30–50 µl of 50% anti-HA (clone HA-7)-coupled agarose beads (Sigma-Aldrich). After thorough washes in Triton lysis buffer, proteins were eluted in Laemmli buffer, heat-denatured for 5 min, and separated on a 12.5% SDS-PAGE gel. After protein transfer onto nitrocellulose membrane (BIO-RAD), specific proteins in cell lysates or immunocomplexes were detected by Western Blot using specific antibodies. For cell cycle analysis and study of protein complexes in condition of endogenous DCAF1 depletion, HEK293T cells were seeded on 6-well plates. Transfections were performed using Lipofectamine 2000 reagent (Invitrogen) according to the manufacturer’s instructions.

### SiRNA-mediated protein depletion

siRNA targeting DCAF1 (siRNA bp3 from the siGENOME SMARTpool, M-021119-03 with the 5′-UCACAGAGUAUCUUAGAGA-3′ sequence targeting the DCAF1 mRNA ORF region 3148-3166) and non-targeting control siRNA (non-targeting siRNA #2) were obtained from Dharmacon. For transfection of HEK293T cells, 80 pmol of siRNA and plasmid DNA constructs encoding HA-tagged Vpr (50 ng) and DCAF1 (250 ng) were preincubated with 4.5 µl of lipofectamine 2000 and overlayed on cells at 50% confluency (final concentration of siRNA was 40 nM). To mark transfected cells, 1 µg of GFP-expressing plasmid (pQBI-25) was usually co-transfected in these experiments. All analyses were conducted at 48 h post-transfection.

### Cell cycle analyses

Cell cycle analysis was performed on the transfected cell population (GFP-positive cells) using propidium iodide (PI) staining as described previously [8], [25]. Briefly, transfected cells were fixed with 1% paraformaldehyde for 15 min, followed by fixation/permeabilization with 70% ethanol for 10 min. The mathematical model MODFIT (Verity Software House) was used to calculate the proportion of cells in the G_2_/M *versus* G_1_ phase of the cell cycle. Statistical analysis was performed using Prism software v. 6.0a (Graph Pad).

### Scanning and quantitation

Bands corresponding to Myc-DCAF1, Myc-DCAF1-WD WT and mutants, endogenous DDB1 and HA-Vpr in the imunoprecipitated fractions were scanned by laser densitometry using an ArtixScanM1 scanner (Microtek). Densitometric quantitation of the bands was performed using the ImageJ software (NIH). Statistical analysis was performed using paired Student’s *t*-tests, and *p* < 0.05 was considered significant as follows: ****: p<0.0001; ***: 0.0001<p<0.001; **: 0.001<p<0.01; *: 0.01<p<0.05; Data were analyzed and plotted using Prism software.

### Secondary structure prediction

A consensus for the secondary structure prediction of DCAF1 WD 1041-1393 was generated using PSI-PRED (Protein Structure Prediction Server) with default settings (http://bioinf.cs.ucl.ac.uk/psipred/) as well as with the secondary structure prediction obtained from the 3D modelization using the LOMETS server (see supporting information).

### 3D modelization

The 3D structure of DCAF1 WD (residues 1041-1393) was modelized using the LOMETS server (on-line web service for protein structure prediction) [26] with its default settings (http://zhanglab.ccmb.med.umich.edu/LOMETS/). This protein structure prediction tool generates 3D models by collecting high-scoring target-to-template alignments from 10 locally-installed threading programs (namely FUGUE, HHsearch, MUSTER, PPA, PROSPECT2, SAM-T02, SPARKS, SP3, FFAS and PRC). The figures of the 3D model were prepared using the PyMOL Molecular Graphics System (DeLano Scientific, Palo Alto, CA, USA).

### Fluorescence Microscopy

Fifty thousand HeLa cells were seeded on cover slips in 24-well plates. Cells were transfected with Lipofectamine 2000 (Invitrogen) according to the manufacturer’s instructions. Two days later, cells were processed for fluorescence immunohistochemistry and laser-scanning confocal microscopy as previously described [27]. Images were acquired using the Zeiss LSM 700 system with ZEN 2010 software and processed using Axio Vision version 4.7.

### Cell fractionation

Cells were lysed in Triton lysis buffer (50 mM Tris pH 7.5, 150 mM NaCl, 0.5% Triton X-100, and a complete protease inhibitors cocktail (Roche)) for 10 min. The fraction containing insoluble cell debris and chromatin was pelleted by centrifugation at 2000 rpm for 10 min. The supernatant was harvested and represented the soluble fraction (S). Chromatin-containing pellets were washed once with benzonase buffer (50 mM Tris pH 8.0, 1.5 mM hydrated MgCl_2_, 0.5% Triton X-100, 0.1 mg/mL BSA and complete protease inhibitors), and re-supended in benzonase buffer containing 0.25 U/mL of benzonase (Stratagene). Pellets were incubated for 30 min to 1h on ice and then centrifuged at 13000 rpm for 15 min. The supernatant was harvested and represented the chromatin-bound fraction (C).

## Results

### DCAF1 WD (1041-1393) interacts with both Vpr and DDB1

**Figure 1:**
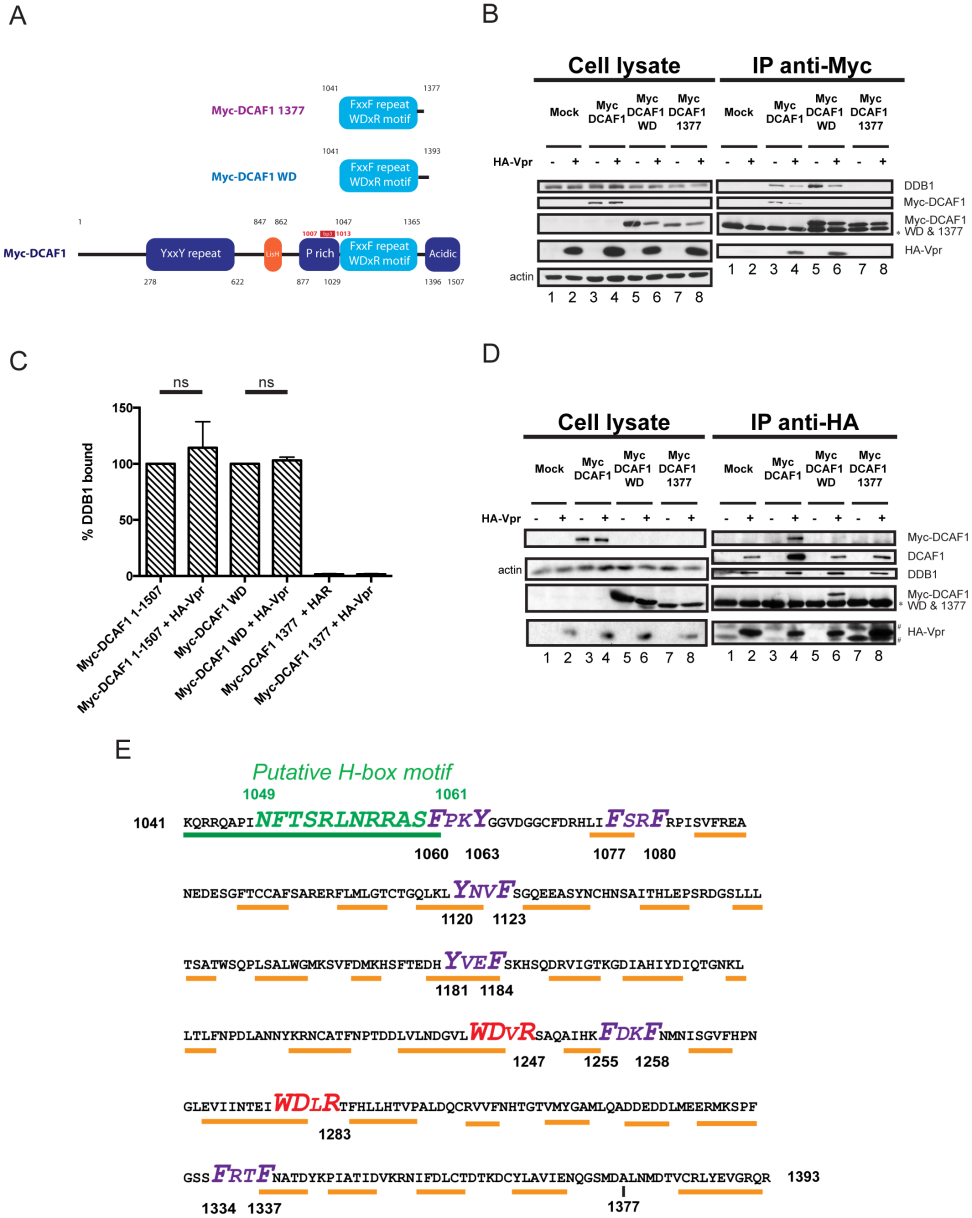
Delineation of the minimal domain of DCAF1 that interacts with HIV-1 Vpr and DDB1. **A.** Schematic representation of Myc-DCAF1 WT (1-1507), Myc-DCAF1 WD (1041-1393) and Myc-DCAF1 1377 (1041-1377). The different domains of DCAF1 with their amino-acid positions are highlighted. Additionally, the region targeted by the full length DCAF1-specific bp3 siRNA is highlighted in red (see below). **B-C**. HEK293T cells were mock-transfected (lanes 1 and 2) or transfected with Myc-DCAF1 (1-1507) (lanes 3 and 4), Myc-DCAF1 WD (1041-1393) (lanes 5 and 6) or with Myc-DCAF1 1377 (1041-1377) (lanes 7 and 8) -encoding plasmids in the presence of empty vector (lanes 1, 3, 5 and 7) or HA-tagged Vpr-expressing plasmid (lanes 2, 4, 6, and 8). Total amounts of DNA were adjusted with empty vector so that similar quantities of plasmids were transfected in each sample. **B.** Immunoprecipitations using anti-Myc antibody were performed on cell extracts using protein-A sepharose beads. The levels of HA-Vpr, endogenous DDB1, Myc-DCAF1 proteins and actin were monitored in cell extracts as well as, when applicable, in immunoprecipitated fractions by Western Blot using specific antibodies. **C.** Quantitation of DDB1 binding efficiency. Band signals corresponding to DDB1 in immunocomplexes were scanned by laser densitometry. The ratio of DDB1 signal over that of precipitated Myc-DCAF1 1507 or Myc-DCAF1WD was calculated and expressed as the percentage of that obtained in the absence of Vpr, which was assigned a value of 100%. Error bars indicate the standard error of the mean (SEM) from the quantitative analysis of three independent experiments. Statistical analysis was performed as described in the Experimental Procedures (p<0.05; ns; non significant). **D**. Immunoprecipitation using anti-HA antibody was performed on cell extracts using anti-HA antibody-coupled agarose beads. The levels of HA-Vpr, endogenous DDB1, endogenous DCAF1, Myc-DCAF1 proteins and actin were monitored in cell extracts as well as, when applicable, in immunoprecipitated fractions by Western Blot using specific antibodies. The data shown here are representative of results obtained in three independent experiments. * denotes the light chain of the IgG used for immunoprecipitation. # represents non-specific immunoprecipitated proteins. **E.** Structural and molecular features of the DCAF1 WD minimal domain. Consensus secondary structure prediction of DCAF1 WD 1041-1393 was generated using the PSI-PRED server and structural data obtained from the 3D modelization. Orange lines highlight the predicted β-sheet structures while the green line and the green amino-acid residues highlight α-helices and the putative H-box motif (see below), respectively. The F/YxxF/Y repeats are highlighted in purple whereas the WDxR motifs are highlighted in red.

The C-terminal region of DCAF1 encompassing the two WD-40 motifs was previously reported to be sufficient for the formation of a ternary complex with Vpr and DDB1 when the three proteins were co-expressed in HEK293T cells [6], [22], [24]. To confirm these results and delineate the minimal domain of DCAF1 required to recruit both Vpr and endogenous DDB1, we tested the ability of three different Myc-tagged DCAF1 variants, including full-length DCAF1 (1-1507), DCAF1 WD (1041-1393) and DCAF1 1377 (1041-1377) (Fig. 1A), to form a ternary complex when co-expressed with HA-Vpr in HEK293T cells. As shown in Figure 1B, HA-Vpr and endogenous DDB1 could be detected in complexes precipitated with Myc-DCAF1 or Myc-DCAF1 WD but not in those pulled-down with Myc-DCAF1 1377 (Fig. 1B, compare lanes 4, 6 and 8), suggesting that Myc-DCAF1 WD 1041-1393 has the ability to bind both Vpr and DDB1 while Myc-DCAF1 1041-1377 does not. A quantitative analysis of the bands revealed that endogenous DDB1 comparably co-precipitated with Myc-DCAF1 or Myc-DCAF1 WD in the presence or the absence of Vpr (compare lanes 3 and 4 as well as lanes 5 and 6), providing further evidence that DCAF1 and DCAF1 WD can engage Vpr and DDB1 simultaneously and form ternary complexes (Fig. 1C). Noticeably, in conditions where Myc-DCAF1 or Myc-DCAF1 WD were expressed, HA-Vpr could not only pull down endogenous DDB1 and Myc-DCAF1 or Myc-DCAF1 WD but also the endogenous DCAF1, indicating that HA-Vpr can form complexes with both the exogenous and endogenous DCAF1’s (Fig. 1D).

Since high resolution structures of DCAF1 or DCAF1/Vpr complexes are currently unavailable, we used the LOMETS server to model the 3D structure of DCAF1 WD from residues 1041 to 1393 (Fig. S1). As shown, the minimal domain of DCAF1 capable of recruiting both Vpr and endogenous DDB1 is predicted to fold as a β-propeller, a structure which is similar to that already described for DDB1 and thought to be involved in protein-protein interactions [28]. Additionally, the N-terminal region of DCAF1 WD is predicted to fold as an α-helix, raising the possibility that this region might contain the H-box motif required for DDB1 binding (Fig. S1 and Fig.1E). Interestingly, besides a putative H-box motif and two adjacent WDxR motifs described to be critical for DCAF interaction with DDB1 [21], [29], the minimal DCAF1 WD domain was found to contain six F/YxxF/Y repeats (where x represents highly variable residues and F/Y represents phenylalanine or tyrosine residues) reminiscent of the WxxF motif (where x represents highly variable residues while W and F represent tryptophan and phenylalanine residues, respectively) previously reported to be critical for Vpr interaction with cellular uracil DNA glycosylase (UNG) and heterologous proteins containing such a motif [30], [31], [32] (Fig. 1E). Thus, the minimal domain of DCAF1 capable of binding both Vpr and DDB1 is predicted to fold as a β-propeller and contains putative motifs for recruitment of both Vpr and DDB1.

### Identification of the H-Box motif in DCAF1 WD

**Figure 2:**
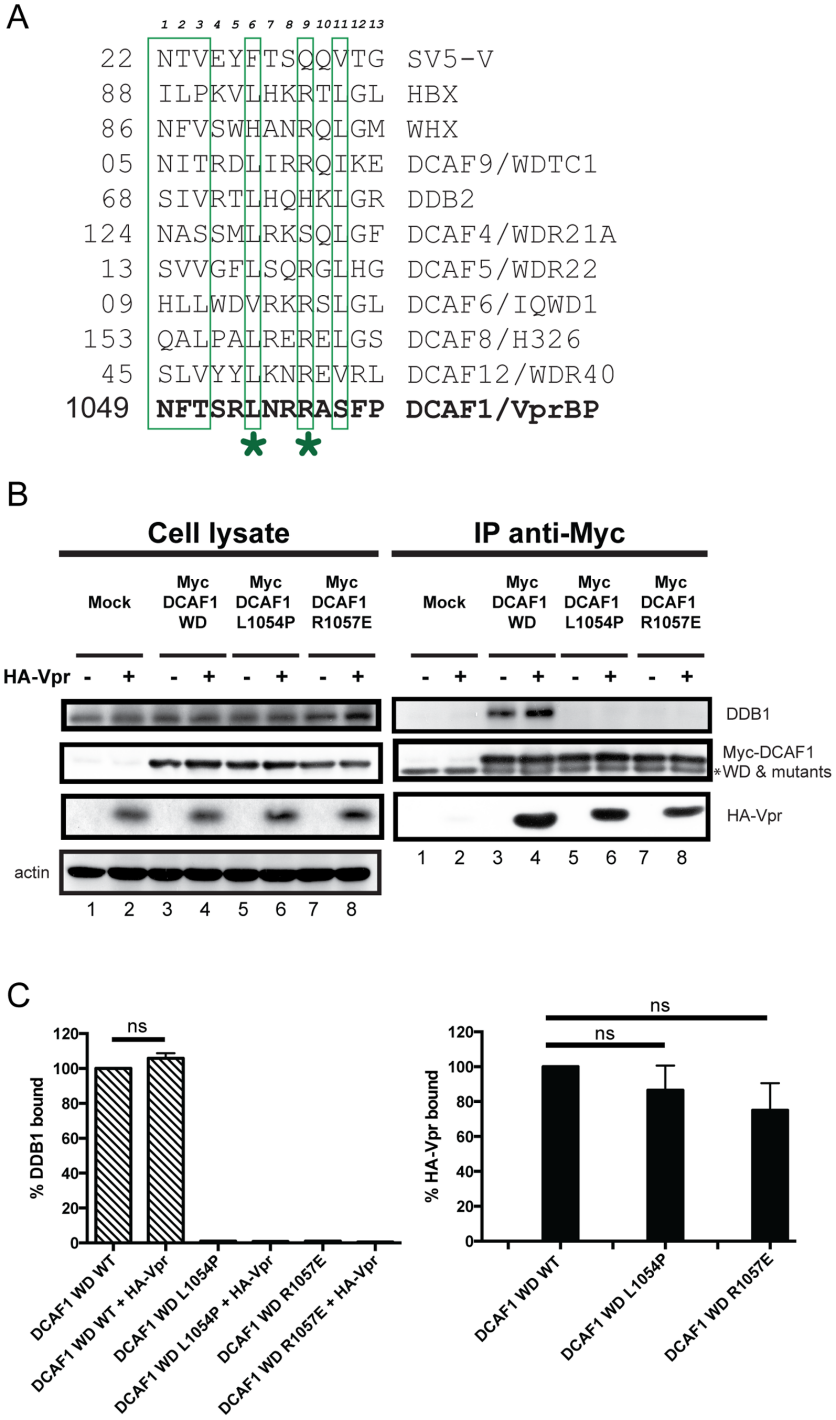
Mutations in the putative H-box motif of DCAF1 abrogate DDB1 binding without affecting Vpr interaction. **A**. Sequence alignment of the H-box motifs of indicated cellular DCAF’s and viral proteins. HBX and WHX indicate viral protein X from Hepatitis B virus and Woodchuck Hepatitis virus, respectively, while SV5-V represents viral protein V encoded by paramyxovirus simian virus 5. Residues of the H-box motif previously reported to contact DDB1 are boxed. Alignment adapted from Li & *al.* [21]. The asterisks indicate the position of substitution mutation (L1054 and R1057). **B.** HEK293T cells were mock-transfected (lanes 1 and 2) or transfected with Myc-DCAF1 WD (lanes 3 and 4), Myc-DCAF1 WD L1054P (lanes 5 and 6) or with Myc-DCAF1 WD R1057E (lanes 7 and 8) -encoding plasmids in the presence of empty vector (lanes 1, 3, 5 and 7) or HA-tagged Vpr-expressing plasmid (lanes 2, 4, 6, and 8). Total amounts of DNA were adjusted with empty vector so that similar quantities of plasmids were transfected in each sample. Immunoprecipitations were performed on cell extracts using anti-Myc antibodies. The levels of HA-Vpr, endogenous DDB1, Myc-DCAF1 WD (WT and mutants) and actin were monitored in cell extracts as well as, when applicable, in immunoprecipitated fractions by Western Blot using specific antibodies. * denotes the light chain of the IgG used for immunoprecipitation. **C.** Quantitation of DDB1 and HA-Vpr binding to Myc-DCAF1 WD. The percentage of DDB1 or HA-Vpr bound to Myc-DCAF1 WD was determined by evaluating the ratio of DDB1 or HA-Vpr band signal over that of Myc-DCAF1 WD WT or mutant in the immunoprecipitated fractions. Ratios obtained with Myc-DCAF1 WD WT were assigned a value of 100%. Error bars indicate the SEM from the quantitative analysis of at least 3 independent experiments. Statistical analysis was performed as described in the Experimental Procedures (p<0.05; ns: non significant).

In order to assess the critical motifs involved in Vpr and/or DDB1 binding, we performed a mutagenic analysis of the different motifs present in DCAF1 WD, and characterized the complexes formed between endogenous DDB1, Vpr and the resulting DCAF1 mutant proteins. Alignment of the DCAF1 putative H-box motif with those previously found in several DCAF’s and viral proteins, such as HBX and SV5-V, revealed a moderate degree of amino-acid conservation at specific positions (Fig. 2A). Conserved leucine and arginine residues at position 1054 and 1057, respectively, were selected for single-point mutation. Leucine 1054 was mutated to proline to completely disrupt the predicted α-helix, while arginine 1057 was substituted for glutamic acid since changing the charge at that position in the HBX H-box motif impaired the recruitment of the DDB1-CRL4A E3 ubiquitin ligase [21]. The data of figure 2 reveals that DCAF1 WD L1054P and DCAF1 WD R1057E completely lost their ability to recruit DDB1 (Fig 2B, lanes 5 and 7 and Fig. 2C), yet they retained the capacity to interact with Vpr (Fig. 2B, compare lanes 4, 6 and 8) almost as efficiently as DCAF WD WT (Fig. 2C). Consistent with the data shown in figure 1, Vpr did not appear to modify the amounts of DDB1 bound to DCAF1 WD (Fig. 2B, lanes 3 and 4, and Fig. 2C) and the presence of Vpr in the mutant DCAF1 WD-containing complexes did not restore any binding to endogenous DDB1 (lanes 6 and 8), thus indicating that DCAF1 acts as a bridge between DDB1 and Vpr within the ternary complex. Taken together, these results suggest that the region spanning residues 1049 to 1061 (NFTSRLNRRASSFP) in DCAF1 is likely to contain the H-box motif required for DDB1 binding and further reveal that the domains of DCAF1 responsible for DDB1 and Vpr binding can be genetically separated.

### Mutation of WDxR motifs abrogates both Vpr and DDB1 binding

**Figure 3:**
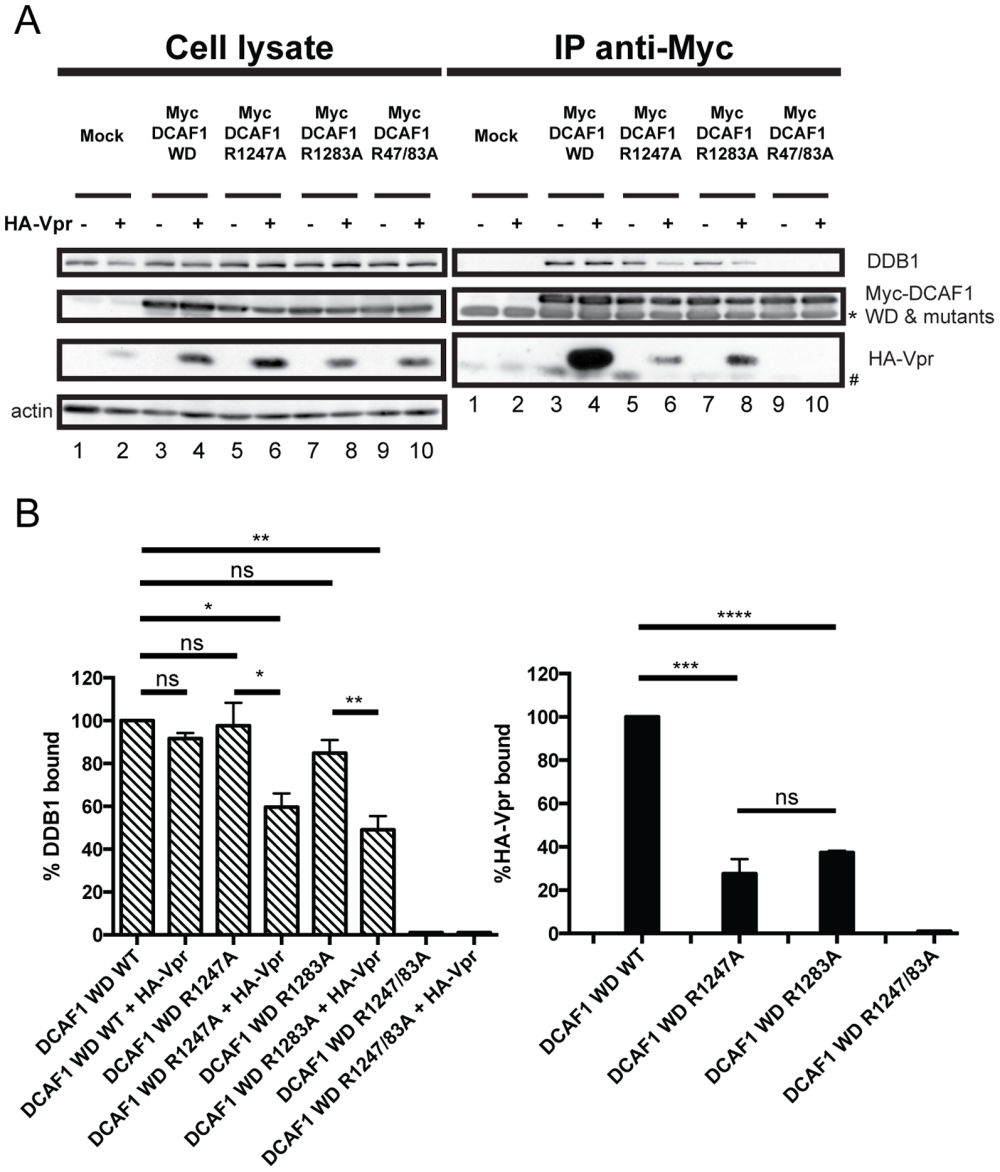
Mutation of DCAF1 WD WDxR motifs differentially affects Vpr and DDB1 binding. **A.** HEK293T cells were mock-transfected (lanes 1, 2) or transfected with Myc-DCAF1 WD (lanes 3 and 4), Myc-DCAF1 WD R1247A (lanes 5 and 6), Myc-DCAF1 WD R1283A (lanes 7 and 8) or with Myc-DCAF1 WD R1247A/R1283A (lanes 9 and 10)-encoding plasmid in the presence of empty vector (lanes 1, 3, 5, 7, and 9) or HA-Vpr expressing plasmid (lanes 2, 4, 6, 8 and 10). Immunoprecipitations and Western Blot detection were performed as described in figure 2B. * denotes the light chain of the IgG used for immunoprecipitation. # represents non-specific immunoprecipitated proteins. **B.** Quantitation of DDB1 and HA-Vpr binding to DCAF1 WD (WT or mutants). Quantitation was determined as described in figure 2C.

The WDxR motifs within DCAF1 were previously reported to be critical for DDB1 binding [29], [33]. Moreover, substitution of the critical arginine residues within the two motifs (arginine 1247 and 1283 were substituted for histidines) resulted in a loss of Vpr interaction**,** most likely as the result of profound changes in the overall folding of DCAF1 [6]. To assess the role of each of the two WDxR motifs in DDB1 and Vpr binding, we mutated each WDxR motif (arginine at positions 1247 or 1283 was substituted for alanine, WDxA) within DCAF1 WD and generated a mutant of DCAF1 WD containing a combination of the two mutated WDxR motifs. DDB1 and Vpr binding was then examined following anti-Myc immunoprecipitation as described in figure 2. As reported by Li and colleagues [33], in the absence of Vpr expression, mutation in individual WDxR motif did not significantly affect DDB1 binding (Fig. 3A, compare lanes 3, 5 and 7, and Fig. 3B), whereas the double mutation completely disrupted DDB1 recruitment (Fig. 3A, compare lanes 3 and 9 and Fig. 3B). In contrast, we observed that mutation of single WDxR motif drastically decreased Vpr interaction, thus highlighting the importance of each of these individual motifs in Vpr recruitment (Fig. 3A, lanes 4, 6 and 8 and Fig. 3B). Interestingly, in these conditions, the diminished Vpr binding observed with single WDxR motif mutants of DCAF1 (60–80% compared to WD WT) was accompanied by a significant reduction of DDB1 binding (40–60% compared to WD WT) (Fig. 3B), suggesting that in the context of this DCAF1 mutant Vpr co-expression affected DDB1 interaction. The double mutant DCAF1 R1247/1283A completely lost its ability to bind both DDB1 and Vpr, most likely due to the overall folding defects of the DCAF1 WD β-propeller (Fig. 3A, compare lanes 4 and 10). These results reveal that Vpr binding to DCAF1 is dependent on the presence of two intact WDxR motifs, while DDB1 binding appears to require that at least one WDxR motif be functional.

### The F/YxxF/Y motifs of DCAF1 are not specifically involved in Vpr binding

**Figure 4:**
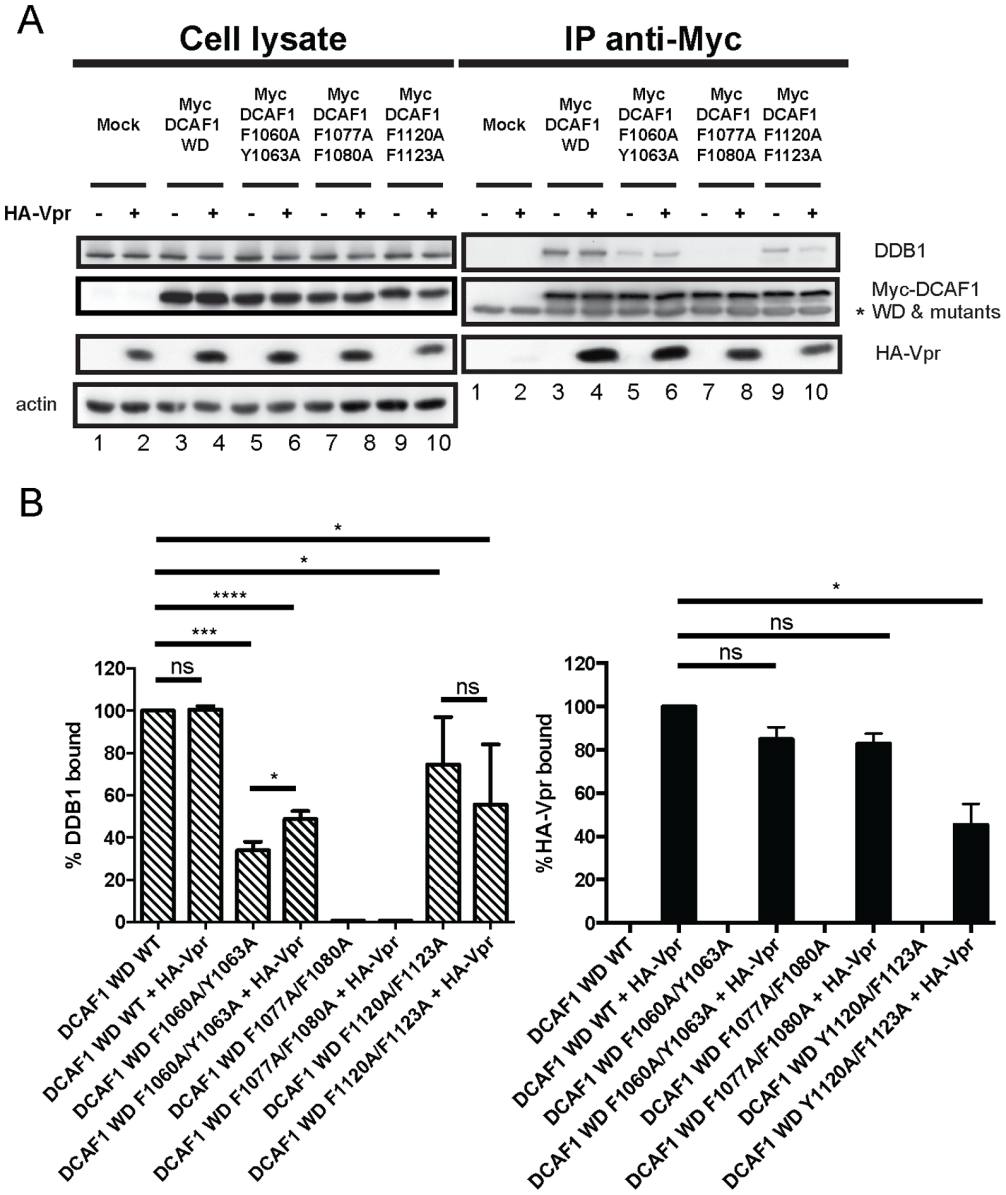
Effect of mutations in the N-terminal F/YxxF/Y motifs of DCAF1 on Vpr and DDB1 binding. **A.** HEK293T cells were mock-transfected (lanes 1 and 2) or transfected with Myc-DCAF1 WD (lanes 3 and 4), Myc-DCAF1 WD F1060A/Y1063A (lanes 5 and 6), Myc-DCAF1 WD F1077A/F1080A (lanes 7 and 8) or with Myc-DCAF1 WD Y1120A/F1123A (lanes 9 and 10)-encoding plasmids in the presence of empty vector (lanes 1, 3, 5, 7 and 9) or HA-Vpr-expressing plasmid (lanes 2, 4, 6, 8 and 10). Immunoprecipitations and Western Blot detection were performed as described in figure 2B. * denotes the light chain of the IgG used for immunoprecipitation. **B.** Quantitation of the DDB1 and HA-Vpr binding. Quantitation was determined as described in figure 2C.

In an attempt to delineate a motif/domain of DCAF1 specifically involved in Vpr recruitment, we mutated the six individual F/YxxF/Y motifs present in DCAF1 minimal domain. To this end, aromatic amino-acid residues (phenylalanine or tyrosine) within each F/YxxF/Y repeat were substituted to alanine since these residues were previously shown to be essential for Vpr binding in the context of WxxF motifs [30]. We first focused on the three F/YxxF/Y motifs located in the N-terminal region of DCAF1 WD (Fig. 1E). Mutations in the Y1120/F1123 motif resulted in a significant reduction of Vpr binding, whereas mutations in the F1060/Y1063 and F1077/F1080 motifs displayed minimal effects at the level of Vpr association (Fig.4A, compare lanes 4 and lanes 6, 8 and 10 and Fig.4B). In contrast, all N-terminal F/YxxF/Y motif DCAF1 mutants harboured a defect in DDB1 recruitment in the absence of Vpr, with DCAF1 WD F1077A/F1080A being the most affected (Fig. 4A, lanes 3, 5, 7 and 9 and Fig. 4B). Indeed, this mutant displayed a phenotype very similar to the H-box motif mutants. Interestingly, although the DCAF1 WD F1060A/Y1063A motif mutant showed a pronounced defect in DDB1 binding (Fig. 4B), this impairment was modestly compensated when the immunoprecipitation was performed in the presence of Vpr (Fig. 4B), suggesting that some cross-talk between Vpr binding and the ability of DCAF1 to recruit DDB1 might indeed occur. This potential DDB1 binding compensation was not observed with DCAF1 WD Y1120A/F1123A as this mutant displayed a significant defect for both Vpr and DDB1 recruitment (Fig. 4A, compare lanes 4 and 10 and Fig. 4B). In fact, similar to the WxDR motif mutants of DCAF1, this F/YxxF/Y motif mutant appeared to display a reduced binding to DDB1 in the presence of Vpr, although statistical significance could not be achieved (Fig. 4B).

**Figure 5:**
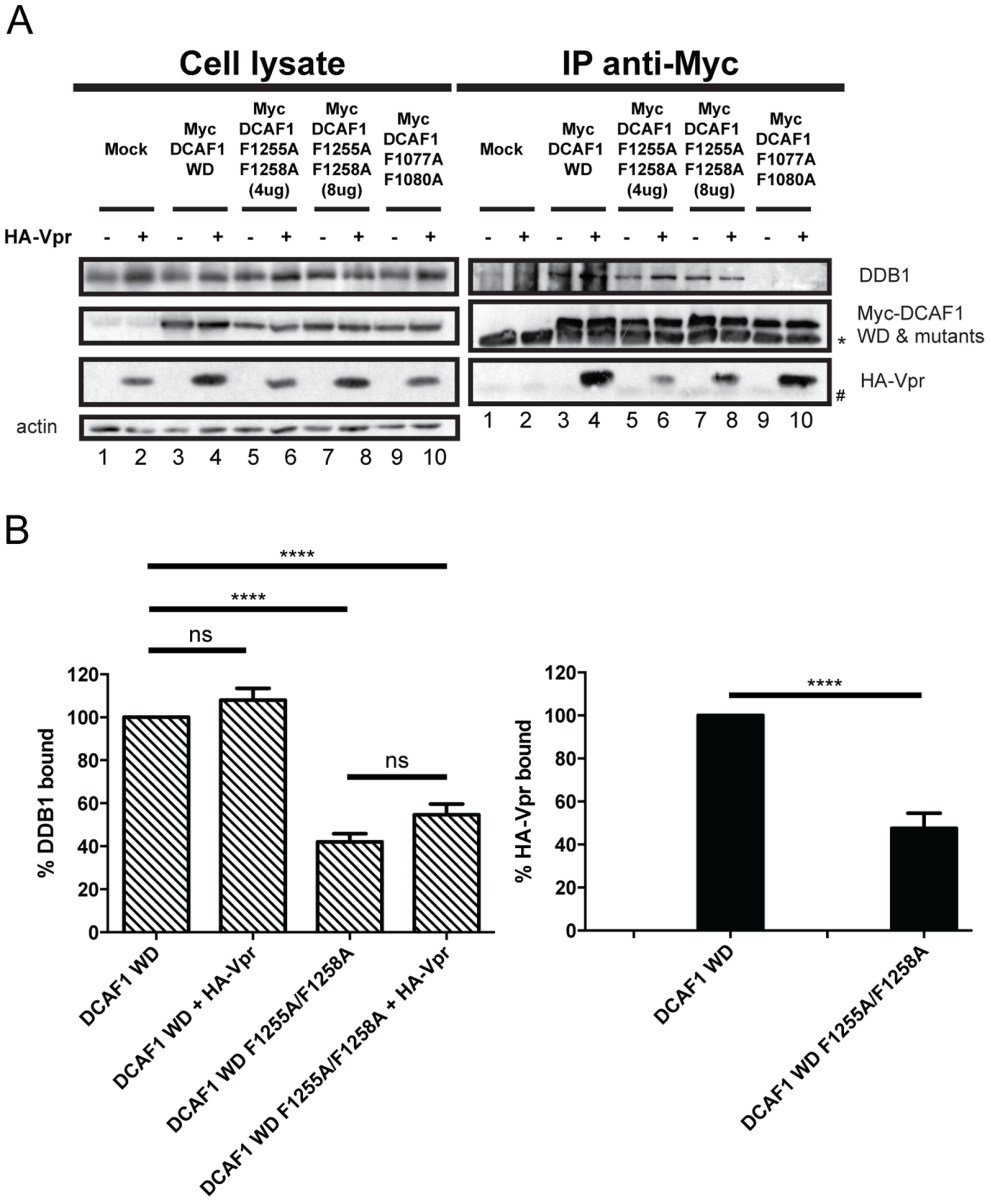
The FDKF motif at position 1255-1258 is not required for efficient recruitment of Vpr. **A.** HEK293T cells were mock-transfected (lanes 1 and 2) or transfected with Myc-DCAF1 WD (lanes 3 and 4), Myc-DCAF1 WD F1255A/F1258A at two different concentrations (lanes 5 to 8), or with Myc-DCAF1 WD F1077A/F1080A (lanes 9 and 10)-encoding plasmid in the presence of empty vector (lanes 1, 3, 5, 7 and 8) or HA-Vpr-expressing plasmids (lanes 2, 4, 6, 8, and 10). Immunoprecipitations and Western Blot detection were performed as described in figure 2B. * denotes the light chain of the IgG used for immunoprecipitation. # represents non-specific immunoprecipitated proteins. **B**. Quantitation of the DDB1 and HA-Vpr binding. Quantitation was determined as described in figure 2C.

We next analyzed the role of the remaining F/YxxF/Y repeats in Vpr and DDB1 binding by mutating the FxxF motif (position 1255-1258) present between the two WDxR motifs as well as the YxxF and FxxF motifs located at positions 1181-1184 and 1334-1337 of DCAF1 WD (Fig.1E and Fig. S2). Mutations in the FxxF motif located between the two WDxR motifs resulted in a significant defect in both Vpr and DDB1 binding (Fig. 5A and B; two concentrations of the Myc-DCAF1 F1255A/F1258A mutant were used to obtain comparable levels of precipitated mutant proteins relative to DCAF1 WD). Interestingly, in the context of this mutant, co-expression of Vpr did not have any detectable effects on DDB1 binding (Fig. 5A, compare lanes 4 and 8 and Fig 5B). The Myc-DCAF1 F1077A/F1080A mutant that is specifically impaired in DDB1 binding was used as a control (lane 9 and 10). Finally, mutations in the F/YxxF/Y motifs of DCAF1 WD at positions 1181-1184 and 1334-1337 resulted in a very strong impairment of both Vpr and DDB1 binding to DCAF1 WD (Fig. S2). Overall, these results indicate that none of the six F/YxxF/Y motifs of DCAF1 are specifically implicated in Vpr binding. Rather they seem to be involved with DDB1 recruitment to DCAF1 in the case of the first two N-terminal motifs or with the overall folding of the protein in the case of the remaining motifs as their alteration interferes with both Vpr and DDB1 binding. Importantly, the increased DDB1 binding observed with the F1060/Y1063 motif mutant of DCAF1, which is fully competent for Vpr binding, suggests that some cross-talk may actually occur between Vpr and DDB1 upon Vpr binding to DCAF1.

### The minimal binding domain of DCAF1 is unable to restore Vpr-mediated G_2_ cell cycle arrest

**Figure 6:**
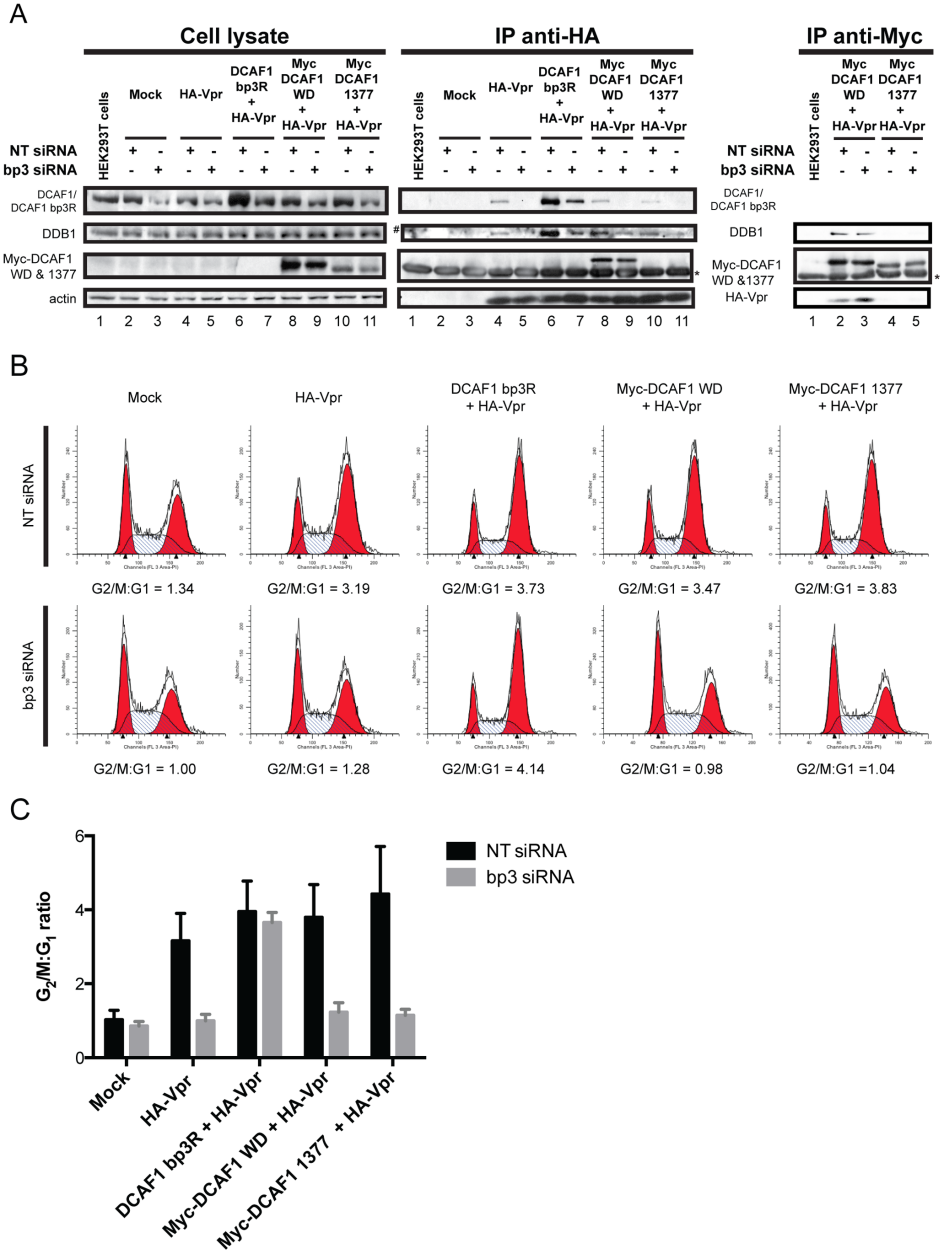
DCAF1 does not solely act as a bridge to engage Vpr to the DDB1-CRL4A E3 ubiquitin ligase and induce G_2_ cell cycle arrest. A-B. HEK293T cells were mock-transfected (lanes 2 and 3) or transfected with HA-Vpr-expressing plasmid (lanes 4 and 5) or transfected with DCAF1 bp3R (DCAF1 1-1507 bp3 siRNA resistant) (lanes 6 and 7), Myc-DCAF1 WD (lanes 8 and 9) or Myc-DCAF1 1377 (lanes 10-11)-encoding plasmids in the presence of HA-Vpr-expressing plasmid. All cells were also transfected with a plasmid encoding GFP and treated with either non-targeting siRNA (*NT siRNA*) (lanes 2, 4, 6, 8 and 10) or specific DCAF1 bp3 siRNA (*bp3 siRNA*) (lanes 3, 5, 7, 9 and 11). Non-transfected HEK293T cells were used as negative control (lane 1). **A.** Non-transfected or transfected HEK293T cells were lysed in 0.5% Triton lysis buffer and subjected to anti-HA or anti-Myc immunoprecipitation and further resolved on SDS-PAGE. The level of HA-Vpr, actin, exogenous and endogenous DCAF1, endogenous DDB1, Myc-DCAF1 WD and Myc-DCAF1 1377 were monitored in cell extracts as well as in the immunoprecipitated fractions by Western Blot using specific antibodies. * denotes the light chain of the IgG used for immunoprecipitation. # represents non-specific immunoprecipitated proteins. **B.** Cell cycle profile of transfected cells (GFP+) analyzed in A. G_2_/M:G_1_ ratios were determined using the Modfit Software. **C.** The graph depicts the mean G_2_/M:G_1_ ratios obtained in two independent experiments. Errors bars represent SEM.

Lastly, we investigated whether DCAF1 is simply acting as a bridge to connect Vpr to the DDB1-CRL4A E3 ubiquitin ligase, or whether the substrate specificity receptor is indeed providing additional functional determinants to enable Vpr-mediated G_2_ cell cycle arrest. A complementation assay was developed in which endogenous DCAF1 was depleted with a specific siRNA (designated bp3, target sequence presented in Fig. 1A), and siRNA-resistant DCAF1-expressing constructs were introduced in *trans.* These included full-length DCAF1 containing point mutation in the siRNA bp3 targeting region (DCAF1 bp3R) as well as Myc-DCAF1 WD and Myc-DCAF1 1377, which do not contain sequences targeted by siRNA bp3. It is important to note, that we had to use an untagged DCAF1 bp3R-encoding construct as a positive control for these experiments since Myc-DCAF1, as opposed to endogenous DCAF1 and Myc-DCAF1 WD, was primarily localized in the cytoplasm and upon co-expression with HA-Vpr redistributed the viral protein from the nucleus to the cytoplasm (Fig. S3A). All DCAF1 constructs expressed in *trans* were minimally affected by siRNA bp3 (note that endogenous DCAF1 and DCAF1 bp3R are indistinguishable in lane 6 and that residual DCAF1 likely representing DCAF1 bp3R is detected in the presence of bp3 siRNA in lane 7) while endogeneous DCAF1 was significantly depleted by the siRNA treatment (∼55–65% depletion) (Fig. 6A). Anti-HA-Vpr immunoprecipitation was performed to examine DCAF1/DDB1/Vpr complex formation in comparable pull-down conditions (Fig. 6A). Consistent with results of figure 1D, complexes were observed between HA-Vpr, endogenous DDB1, endogenous DCAF1 and with the exogenously-expressed DCAF1 bp3R and Myc-DCAF1 WD, but not with Myc-DCAF1 1377 (Fig. 6A, compare lanes 4, 6, 8 and 10 IP anti-HA), in non-siRNA targeting conditions. As expected, in conditions where endogenous DCAF1 was depleted, complexes comprising of HA-Vpr, endogenous DDB1, DCAF1 bp3R or Myc-DCAF1 WD were reconstituted (Fig. 6A, lanes 7 and 9 of IP anti-HA) while those with Myc-DCAF1 1377 were not (Fig. 6A, lane 11 IP anti-HA). Additionally, anti-Myc immunoprecipitation was performed to confirm that Myc-DCAF1 WD was indeed forming a complex with HA-Vpr and DDB1 in both non-targeting and siRNA bp3 targeting conditions while Myc-DCAF1 1377 did not (Fig. 6A, lanes 2-5, IP anti-Myc). To assess whether the exogenous DCAF1-containing complexes formed were functionally active, cell cycle analysis was carried-out in parallel (Fig. 6B-C). As previously reported [3], [4], [5], [6], [7], [8], [9], depletion of endogenous DCAF1 impaired the formation of a Vpr-DDB1-DCAF1 complex (Fig. 6A compare lanes 4 and 5) and inhibited the G_2_ cell cycle arrest induced by Vpr (Fig. 6B; compare G_2_/M:G_1_ ratio of 3.19 to 1.28 and Fig. 6C). Interestingly, complementation experiments with a DCAF1 protein resistant to the bp3 siRNA restored the formation of a Vpr-DCAF1-DDB1 complex (Fig. 6A, compare lanes 5 and 7) and re-established a Vpr-induced G_2_ arrest of the same magnitude to that observed in non-targeting siRNA conditions (Fig. 6B, compare G_2_/M:G_1_ ratio of 3.73 to 4.14 and Fig. 6C). In contrast, complementation with the minimal domain of DCAF1, which is capable of binding both Vpr and DDB1 (Fig. 6A, compare lanes 8 and 9 of IP anti-HA and lanes 2 and 3 of IP anti-Myc), failed to restore the Vpr-mediated cell cycle block (Fig. 6B, compare G_2_/M:G_1_ ratio of 3.73 to 0.98 and Fig. 6C). Importantly, we confirmed that this complex was not G_2_ arrest-incompetent due to cellular mislocalization since both Vpr and Myc-DCAF1 WD were found to co-localize in the nucleus (Fig. S3A). Moreover, similar to endogenous DCAF1, a fraction of Myc-DCAF1 WD was indeed associated to the chromatin (Fig. S3B). As expected, Myc-DCAF1 1377, which did not form a complex with Vpr and DDB1 (Fig. 6A, lanes 10 and 11 of IP anti-HA, lanes 4 and 5 of IP anti-Myc), failed to restore any Vpr-mediated cell cycle arrest as the G_2_/M:G_1_ ratio remained equivalent to the mock control (Fig. 6B, compares 1.04 to 1.00; and Fig. 6C). Overall, our results suggest that the minimal domain of DCAF1, which contains the motifs required for proper recruitment of both Vpr and DDB1, is not sufficient to support Vpr-mediated G_2_ arrest activity.

## Discussion

In this study, we sought to identify molecular and structural determinants of DCAF1 that are crucial for DDB1 and/or Vpr binding to better understand the architecture of the putative DDB1-DCAF1-Vpr substrate-recognition module. Furthermore, we examined whether DCAF1 acted solely as an adaptor to bridge Vpr onto the DDB1-CUL4A complex, or whether DCAF1 was providing additional functions that would be central to Vpr-mediated G_2_ arrest. First, we showed that a region of DCAF1 extending from residues 1041 to 1393 and predicted to fold as a β-propeller (DCAF1 WD) represents the minimal domain capable of binding both Vpr and DDB1 (Fig.1 and Fig. S1). The minimal domain delineated in our study is slightly longer than the one reported by Le Rouzic and colleagues, which indeed encompassed the region between residues 1041 and 1377 [6]. In fact, analysis of a similar DCAF1 domain (DCAF1 1377) revealed that this fragment was unable to bind Vpr and DDB1 (Fig.1 and Fig. 6) most likely because it lacked a small region predicted to form a β-sheet structure (residues 1384-1392, Fig. 1E), a condition that might affect the protein folding as a β-propeller. Interestingly, the minimal domain of DCAF1 targeted by Vpr does not comprise the region required for interaction with the cellular protein Merlin (neurofibromin 2) or the human cytomegalovirus CMV UL35 protein, two proteins that appear to negatively regulate the CRL4A (DCAF1) E3 ligase activity by interacting with the C-terminal acidic region of DCAF1 [33], [34], [35]. Thus, the fact that Vpr engages the CRL4A (DCAF1) E3 ligase through a domain of DCAF1 that is distinct from that targeted by putative negative regulators provides further evidence that Vpr-mediated G_2_ cell cycle arrest is unlikely to involve a suppression of the CRL4A E3 ligase activity.

Sequence alignment of viral proteins and DCAFs known to interact with DDB1 combined to mutagenesis allowed us to delineate the putative H-box motif of DCAF1 within an α-helical region encompassing amino-acid residues 1049 and 1061. Indeed, mutation that were previously reported to impair the H-box motif of the X protein of HBV [21], resulted in a very strong impairment of DDB1 binding without significantly affecting Vpr binding (Fig. 2). While the identification of the DCAF1 H-box motif confirms and extends the findings of Li and colleagues [21], it also revealed that the domains of DCAF1 responsible for DDB1 and Vpr binding can be genetically separated. This notion is also supported by our results showing a differential requirement of DCAF1 WD-40 motifs for Vpr and DDB1 binding (Fig. 3). While the binding of DDB1 to DCAF1 was not significantly affected when individual WD-40 motifs were mutated, binding of Vpr to DCAF1 required that both WD-40 be intact, suggesting that this interaction was more dependent on the conformation of DCAF1. Several lines of evidence suggest that H-box motifs are not the only structural determinants allowing contact of DCAFs to DDB1. In fact, it was proposed that DCAFs might interact with DDB1 via multiple interfaces, which involve not only the H-box motif but also other parts of the protein most likely the WD-40-repeat motif [21]. In that regard, our mutagenesis of the DCAF1 H-box motif and WD-40-repeats re-emphasizes how DCAF1 might interact with DDB1 through a bipartite binding mechanism that relies on two main determinants: the H-box motif as well as the β-propeller structural determinants conferred through the WD-40 motif [21], [29] (Fig. 2 and 3). Indeed, as previously proposed, one functional advantage of such a bipartite interface would be to provide a unique mechanism for the DDB1-CUL4A (DCAF1) E3 complex to switch between productive and nonproductive forms of an E3 machinery without completely disassembling the ubiquitin ligase complex [17], [21], [29]. Whether Vpr association with DCAF1 would switch the equilibrium towards a productive form of the CRL4A (DCAF1) E3 ubiquitin ligase and thus make Vpr a positive regulator of the CRL4A (DCAF1) E3 ligase activity remains a possibility. In that regard, it is interesting to note that in the context of the DCAF1WD F1060/Y1063A mutant, impaired DDB1 binding was compensated by Vpr association, suggesting potential cross-talk between Vpr binding to DCAF1 and the ability of the substrate specificity receptor to recruit DDB1 (Fig. 4). Additionally, in the context of other mutants (DCAF1 WD R1247A, DCAF1 WD R1283A and perhaps DCAF1 WD Y1120A/F1123A), co-expression of Vpr appeared to affect the binding of DDB1 to DCAF1 (Fig. 3 and 4). Whether this phenotype results from the residual or transient interaction of Vpr in the context of this mutant or to a more efficient recruitment of DDB1 to complexes formed by endogenous DCAF1 and Vpr under these conditions remains an open question. Nevertheless, these observations suggest that co-expression or/and binding of Vpr to DCAF1 appears to modulate the formation of a DDB1/DCAF1 complex.

While it was possible to identify mutants of DCAF1 that lost the ability to bind DDB1 but still able to retain Vpr association (Fig. 2 and 4), we could not identify DCAF1 mutants with the opposite phenotype. Mutation of F/YxxF/Y repeats reminiscent of WxxF motifs present in the Vpr binding partner, UNG, did not reveal any exclusive impairment of Vpr binding (Fig. 4 and 5 and Fig. S2). Indeed, apart from the first two N-terminal F/YxxF/Y repeats, all others appeared involved in the overall structure of DCAF1 since introduction of mutations affected both Vpr and DDB1 binding. While these results did not reveal a specific region of DCAF1 involved in Vpr binding, they nevertheless re-emphasized that the overall conformation of DCAF1 is likely to be important for Vpr binding.

Lastly, we documented that DCAF1 is unlikely to only act as an adaptor to bridge Vpr to the DDB1-CUL4A complex. Indeed, our complementation assay revealed that DCAF1 WD is not sufficient to restore Vpr-mediated cell cycle arrest despite the fact that: 1) complexes between DDB1, DCAF1 WD and Vpr are detected in conditions where endogenous DCAF1 is depleted (Fig. 6); 2) DCAF1 WD and Vpr are co-localized in the nucleus (Fig. S3A); and 3) DCAF1 WD is found associated to the chromatin as endogenous DCAF1 (Fig. S3B). These results imply that DCAF1 provides additional functions to the Vpr/E3 ligase complex that are essential for the induction of a G_2_ cell cycle arrest. While the full-length DCAF1, which is functional in our complementation assay, consists of 1507 amino acid residues and is known to oligomerise through the LisH motif, the DCAF1 WD minimal domain is about 350 amino acids in length and misses this motif. Given that oligomerisation of DCAF1 appears important for enhancing the functional activity of the CRL4A (DCAF1) E3 ligase *in vitro* [23], it is therefore possible that the inability of DCAF1 WD to oligomerize may explain its failure to restore Vpr-mediated G_2_ cell cycle arrest. Moreover, the distance between the substrate and the E2 ligase module being critical for efficient ubiquitination, it is also quite possible that when the CUL4-DDB1 (DCAF1WD)/Vpr complex is reconstituted, this distance is not optimal, thus preventing efficient ubiquitination of substrates targeted by Vpr. Alternatively, we cannot exclude that Vpr usurps the E3 ubiquitin ligase to enhance the degradation of a natural substrate of DCAF1. It may be that DCAF1 WD is unable to recruit the said natural substrate, and consequently in that context Vpr cannot activate the ATR signaling pathway to trigger a G_2_ cell cycle arrest. Cellular substrate(s) normally targeted by DCAF1 in the context of CRL4A have recently been identified and now include Mcm10, a replication factor that plays an essential role in the initiation and elongation of DNA replication [36], perhaps, the tumor suppressor Merlin [34], and recently the uracil DNA glycosylases, UNG2 (nuclear uracil-DNA glycosylase 2) and SMUG1 (single-strand-selective monofunctional uracil-DNA glycosylase), two proteins previously shown to be targeted by HIV-1 Vpr but not linked to Vpr-mediated G_2_ arrest [37], [38], [39]. Interestingly, it was previously reported that UNG2 assembles with and is turned over through the CRL4A (DCAF1) complex in the absence of Vpr but that Vpr enhanced the recruitment of UNG2 to the E3 ligase complex [39].

## Conclusions

Overall, we provide evidence that additional determinants in DCAF1 are crucial for Vpr-mediated G_2_ cell cycle arrest, as a truncated DCAF1 that retains both Vpr and DDB1 binding is not sufficient to restore this activity. Interestingly, since Vpr seems to influence the formation of a complex between DDB1 and DCAF1, it is possible that Vpr modulates the E3 ubiquitin ligase activity to promote ATR activation, and consequently G_2_ cell cycle arrest. Indeed, this manipulation of the CRL4A (DCAF1) E3 ligase would change its activity leading to the enhanced degradation of a natural substrate of DCAF1, rather than redirecting the specificity of the E3 ubiquitin ligase as has been described for other HIV-1 viral accessory proteins hijacking host E3 ubiquitin ligases [40]. However, identification of the factor(s) targeted by the Vpr/CRL4A-DDB1 (DCAF1) E3 ubiquitin ligase complex to trigger G_2_ arrest will be necessary to fully understand the architecture of the complex and the role of Vpr in the life cycle of HIV-1.

## Supporting Information

Figure S1 **3D modelization of DCAF1 WD.** Ribbon diagram of the 3D modelization of DCAF1 WD using the LOMETS server (http://zhanglab.ccmb.med.umich.edu/LOMETS/). The MUSTER sofware (MUlti-Sources ThreadER), which is a protein threading algorithm to identify the template structures from the PDB library, allowed us to generate the best model with a confidence score of 0.929, based on the 3D structure of the WD domain-containing 40S ribosomal protein, RACK1 (pdb code: 3iz6.a) [41]. The best model is represented with orange arrows highlighting the predicted β-sheet and in green the predicted α-helices (Pymol). The F/YxxF/Y repeats and WDxR motifs are highlighted in purple and red, respectively.
(TIF)

Figure S2 **Mutagenesis of F/YxxF/Y repeats at position 1181/1184 and 1334/1337 of DCAF1 disrupts both DDB1 and Vpr binding.** HEK293T cells were mock-transfected (lanes 1 and 2) or transfected with Myc-DCAF1 WD (lanes 3 and 4), Myc-DCAF1 WD Y1181A/F1184A (lanes 5 and 6) or with Myc-DCAF1 WD F1334A/F1337A (lanes 7 and 8)-encoding plasmids in the presence of empty vector (lanes 1, 3, 5 and 7) or HA-Vpr-expressing plasmid (lanes 2, 4, 6, and 8). Immunoprecipitations were performed on cell extracts using anti-Myc antibodies. The levels of HA-Vpr, endogenous DDB1, Myc-DCAF1 WD (WT and mutants) and actin were monitored in cell extracts as well as, when applicable, in immunoprecipitated fractions by Western Blot using specific antibodies. * denotes the light chain of the IgG used for immunoprecipitation. # represents non-specific immunoprecipitated proteins.
(TIF)

Figure S3 **Analysis of Myc-DCAF1 WD localization and association to chromatin.** **A**. HeLa cells were transfected with HA-Vpr-expressing plasmid alone or co-transfected with Myc-DCAF1 WD or Myc-DCAF1 1-1507-encoding plasmids. Forty-eight hours post-transfection, cells were fixed, permeabilized, and stained with antibodies against DCAF1 (green), HA (red), and Myc (magenta). DAPI (4,6-diamidino-2-phenylindole) was used to highlight the nuclei (blue). Images were acquired by confocal microscopy with a 63X objective. Image shown are representative of multiple fields. Merged field present the localization of Vpr and Myc-tagged DCAF1s. **B.** HEK293T were transfected with empty vector or Myc-DCAF1 WD. Forty-eight hours post-transfection, cells were harvested in Triton lysis buffer and subjected to subcellular fractionation. The chromatin-containing insoluble fraction was subjected to benzonase treatment to release chromatin-associated factors. The level of endogenous DCAF1, GAPDH, Histone H3, Myc-DCAF1 WD were monitored in soluble (S) as well as in the nuclease-treated fractions (C) by Western Blot using specific antibodies. Results are representative of 3 independent experiments.
(TIF)

Table S1 **List of primers used to construct the pCS2-myc6-DCAF1 WD mutants using site-directed mutagenesis.**
(TIF)
