# Supplementary figures and images for: Defining the Interactions and Role of DCAF1/VPRBP in the DDB1-Cullin4A E3 Ubiquitin Ligase Complex Engaged by HIV-1 Vpr to Induce a G2 Cell Cycle Arrest
Source: PLoS One. 2014 Feb 18;9(2):e89195. doi: 10.1371/journal.pone.0089195 (PMC3928422; doi:10.1371/journal.pone.0089195)

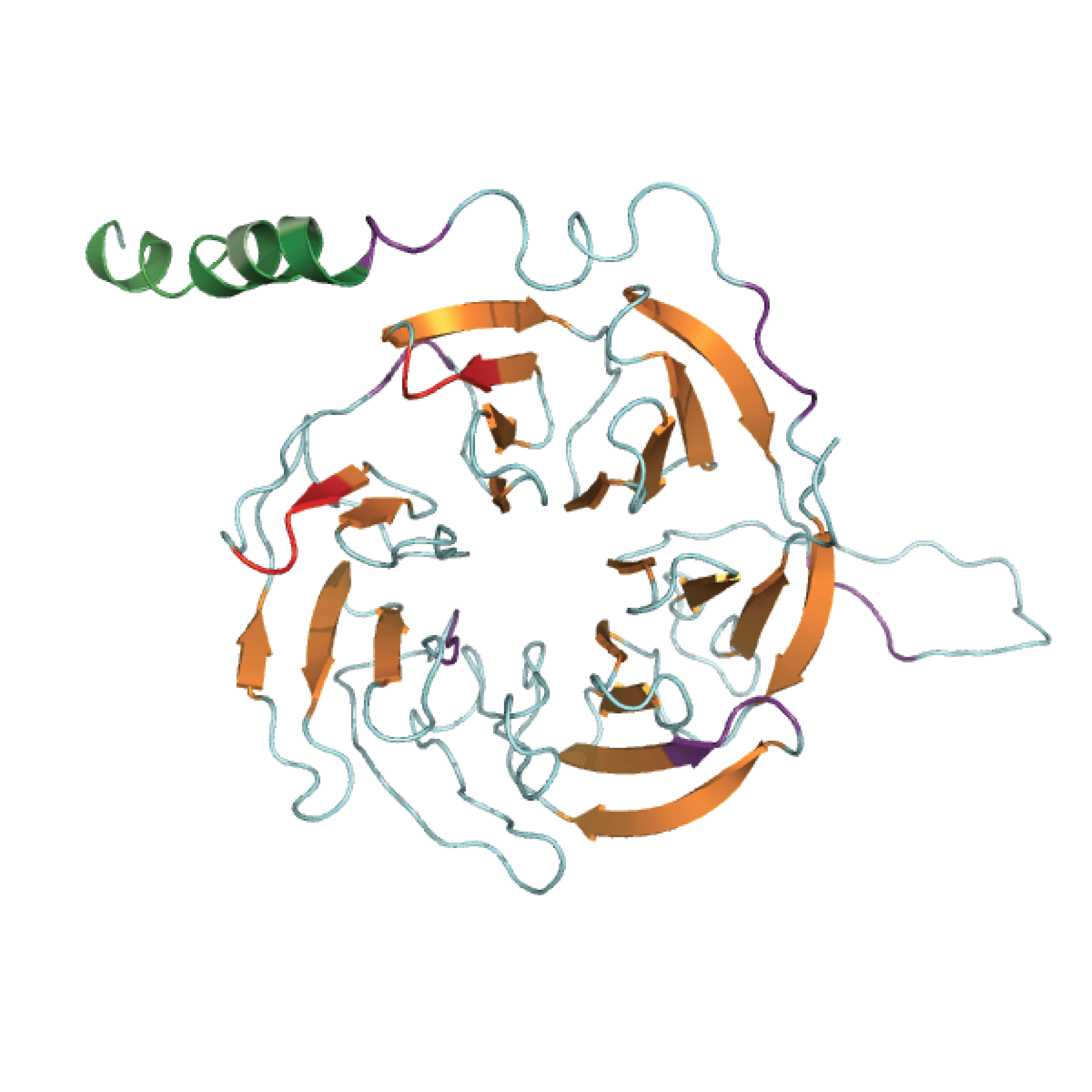

Supplement: Figure S1 — 3D modelization of DCAF1 WD. Ribbon diagram of the 3D modelization of DCAF1 WD using the LOMETS server (http://zhanglab.ccmb.med.umich.edu/LOMETS/). The MUSTER sofware (MUlti-Sources ThreadER), which is a protein threading algorithm to identify the template structures from the PDB library, allowed us to generate the best model with a confidence score of 0.929, based on the 3D structure of the WD domain-containing 40S ribosomal protein, RACK1 (pdb code: 3iz6.a) [41]. The best model is represented with orange arrows highlighting the predicted β-sheet and in green the predicted α-helices (Pymol). The F/YxxF/Y repeats and WDxR motifs are highlighted in purple and red, respectively. (TIF) [file pone.0089195.s001.tif]

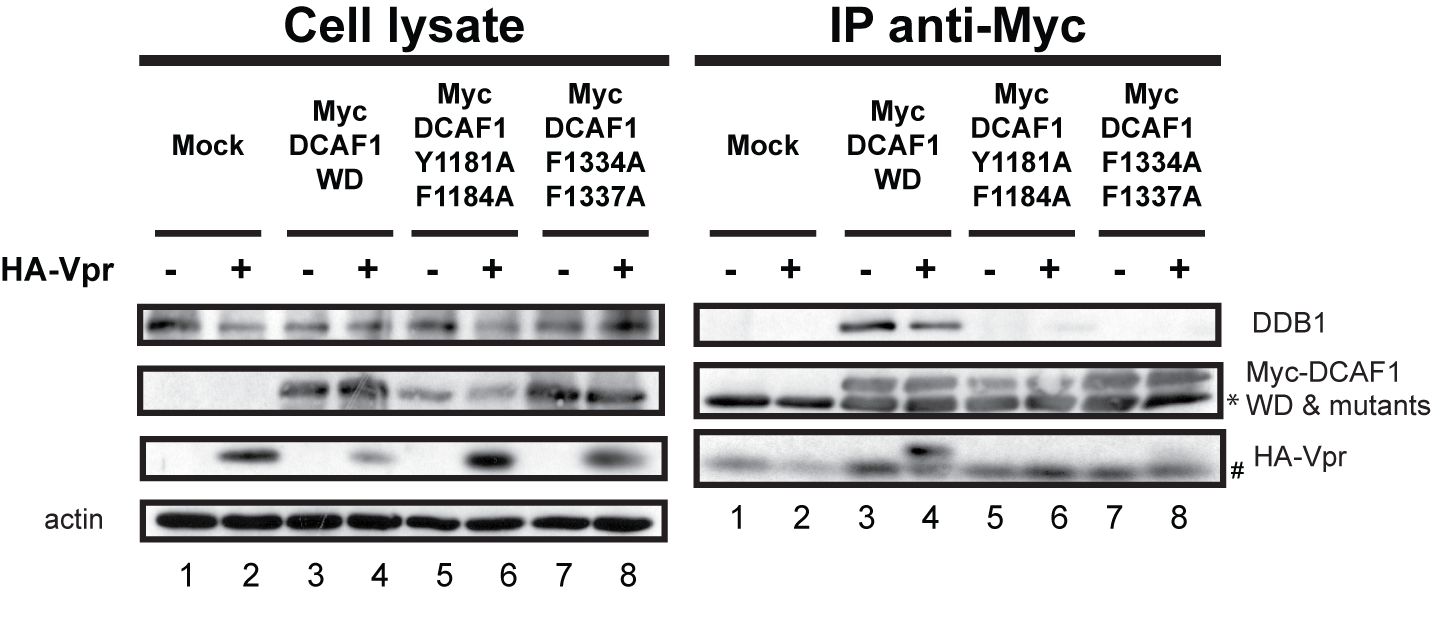

Supplement: Figure S2 — Mutagenesis of F/YxxF/Y repeats at position 1181/1184 and 1334/1337 of DCAF1 disrupts both DDB1 and Vpr binding. HEK293T cells were mock-transfected (lanes 1 and 2) or transfected with Myc-DCAF1 WD (lanes 3 and 4), Myc-DCAF1 WD Y1181A/F1184A (lanes 5 and 6) or with Myc-DCAF1 WD F1334A/F1337A (lanes 7 and 8)-encoding plasmids in the presence of empty vector (lanes 1, 3, 5 and 7) or HA-Vpr-expressing plasmid (lanes 2, 4, 6, and 8). Immunoprecipitations were performed on cell extracts using anti-Myc antibodies. The levels of HA-Vpr, endogenous DDB1, Myc-DCAF1 WD (WT and mutants) and actin were monitored in cell extracts as well as, when applicable, in immunoprecipitated fractions by Western Blot using specific antibodies. * denotes the light chain of the IgG used for immunoprecipitation. # represents non-specific immunoprecipitated proteins. (TIF) [file pone.0089195.s002.tif]

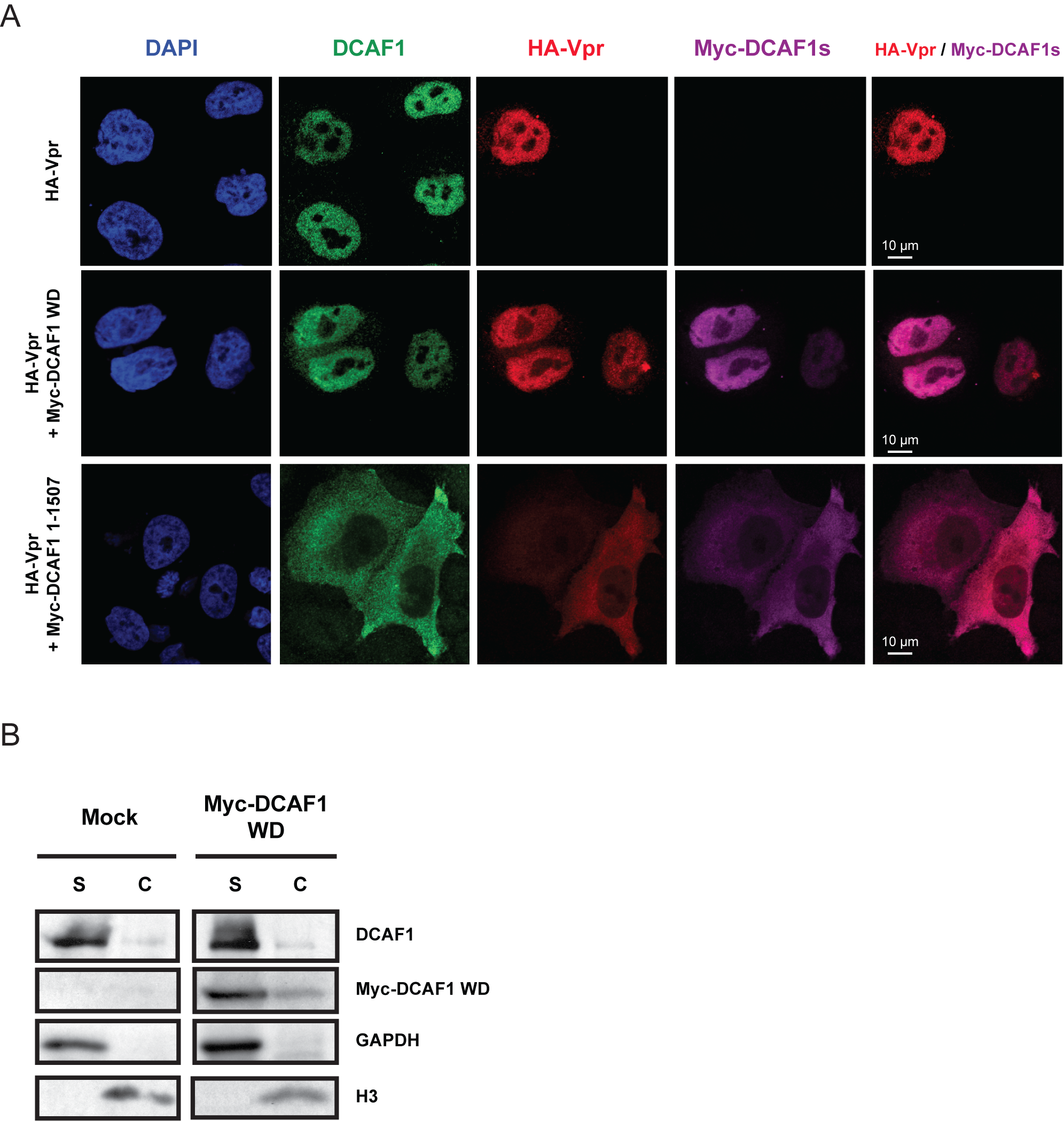

Supplement: Figure S3 — Analysis of Myc-DCAF1 WD localization and association to chromatin. A. HeLa cells were transfected with HA-Vpr-expressing plasmid alone or co-transfected with Myc-DCAF1 WD or Myc-DCAF1 1-1507-encoding plasmids. Forty-eight hours post-transfection, cells were fixed, permeabilized, and stained with antibodies against DCAF1 (green), HA (red), and Myc (magenta). DAPI (4,6-diamidino-2-phenylindole) was used to highlight the nuclei (blue). Images were acquired by confocal microscopy with a 63X objective. Image shown are representative of multiple fields. Merged field present the localization of Vpr and Myc-tagged DCAF1s. B. HEK293T were transfected with empty vector or Myc-DCAF1 WD. Forty-eight hours post-transfection, cells were harvested in Triton lysis buffer and subjected to subcellular fractionation. The chromatin-containing insoluble fraction was subjected to benzonase treatment to release chromatin-associated factors. The level of endogenous DCAF1, GAPDH, Histone H3, Myc-DCAF1 WD were monitored in soluble (S) as well as in the nuclease-treated fractions (C) by Western Blot using specific antibodies. Results are representative of 3 independent experiments. (TIF) [file pone.0089195.s003.tif]

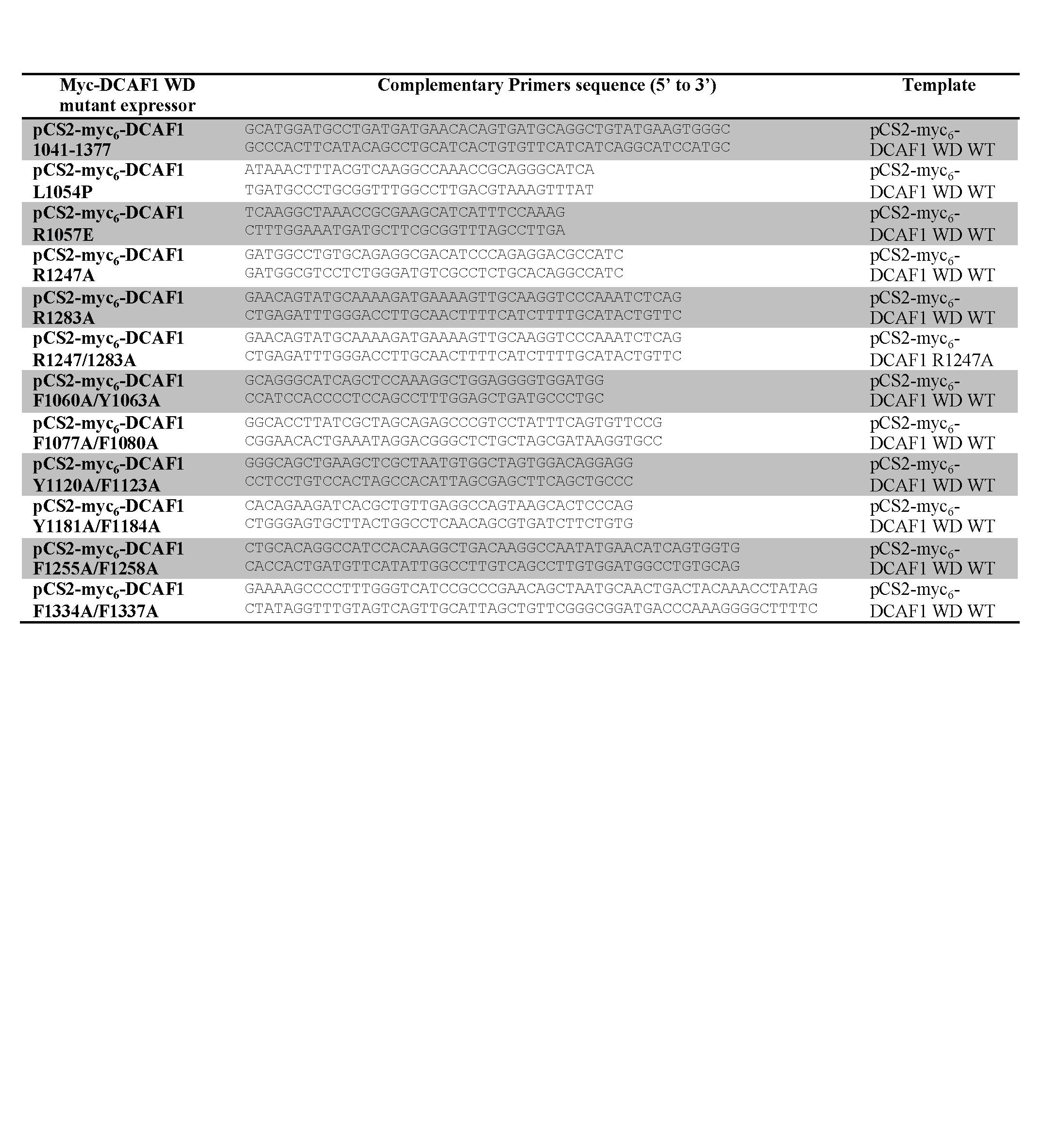

Supplement: Table S1 — List of primers used to construct the pCS2-myc6-DCAF1 WD mutants using site-directed mutagenesis. (TIF) [file pone.0089195.s004.tif]
